# Epigenetic suppression of the anti-aging gene *KLOTHO* in human prostate cancer cell lines

**DOI:** 10.1080/19768354.2017.1336112

**Published:** 2017-06-22

**Authors:** Minkyu Seo, Min Su Kim, Ara Jang, Hyun Joo Chung, Yoohun Noh, Do-Hee Kim, Jaehyouk Lee, Kisung Ko, Soon Chul Myung

**Affiliations:** a Department of Urology, Chung-Ang University College of Medicine, Seoul, Republic of Korea; b Department of Urology, Seoul Medical Center, Seoul, Republic of Korea; c Advanced Urogenital Diseases Research Center, Chung-Ang University College of Medicine, Seoul, Republic of Korea; d Bio-Integration Research Center for Nutra-Pharmaceutical Epigenetics, Chung-Ang University, Seoul, Republic of Korea; e Department of Anatomy and Cell Biology and Neurology, College of Medicine, Chung-Ang University, Seoul, Korea; f Famenity Biomedical Research Center, Famenity, Inc., Gyeonggi, Korea; g Natural Pharmaceutical R&D Center, Naturesense, Inc., Gyeonggi, Korea; h Department of Medicine, College of Medicine, Chung-Ang University, Seoul, Republic of Korea

**Keywords:** Prostate cancer, aging, *KLOTHO*, epigenetic inactivation, DNA methylation

## Abstract

*KLOTHO* was originally identified as an aging-suppressor gene that causes a human aging-like phenotype when tested in *KLOTHO*-deficient-mice. Recent evidence suggests that *KLOTHO* functions as a tumor suppressor by inhibiting *Wnt* signaling. *KLOTHO* gene silencing, including DNA methylation, has been observed in some human cancers. Aberrant activation of *Wnt* signaling plays a significant role in aging, and its silencing may be related to prostate cancer and other types of cancers. Thus, we investigated whether the expression of the anti-aging gene *KLOTHO* was associated with epigenetic changes in prostate cancer cell lines. *KLOTHO* mRNA was detected in the 22Rv1 cell line while it was not detected in DU145 and PC-3 cell lines. The restoration of *KLOTHO* mRNA in the DU145 and PC-3 cell lines was induced with a DNA methyltransferase inhibitor. Methylation-specific PCR was performed to determine the specific CpG sites in the *KLOTHO* promoter responsible for expression. In addition, the level of methylation was assessed in each CpG by performing bisulfite sequencing and quantitative pyrosequencing analysis. The results suggested a remarkable inverse relationship between *KLOTHO* expression and promoter methylation in prostate cancer cell lines.

## 1. Introduction

Prostate cancer is the most common cancer type in American men and is ranked second among the causes of cancer-related deaths (Jemal et al. 2010). In Korea, it is the fifth most commonly diagnosed disease and the seventh most common cause of mortality, indicating a smaller risk compared to that in the United States. However, the social importance of prostate cancer has increased, as the number of patients from 1999 to 2012 increased fivefold and the annual mortality rate also increased (Jung et al. 2015). The known causes for prostate cancer include aging, race, environment, and genetic factors. Among these, aging is assumed to be one of the main causes (Rodriguez et al. 1997; Kwabi-Addo et al. 2007; Yoon & Byun 2010).

Previous studies have suggested that cell aging causes cancer. Some argue that cell aging causes p53 protein modifications related to either apoptosis or cell cycle suppression in injured cells during repetitive cell division cycles (Zuckerman et al. 2009). Others claim that immunosenescence, which refers to suppressed immune function due to natural age advancement, inhibits the recognition of cancer in the early stages, resulting in the onset or relapse of cancer (Coppe et al. 2010). It has been proved that aged cells secrete vascular endothelial growth factor and inflammatory cytokines more than young ones, and these bioactive substances may promote changes in the microenvironment around the aged cell, leading to growth and metastasis of cancer cells (Fulop et al. 2010). However, the relationship between cancer and aging has not been clarified.

*KLOTHO* is a potential gene associated with aging that is usually expressed in distal convoluted tubules in the kidney and the choroid plexus in the brain. Some studies suggested that *KLOTHO*-deficient mice have a phenotype similar to the aging process in humans; they have a life span about 8–9 weeks, which is much shorter than the average of 24 months (Kuro-o et al. 1997). On the other hand, studies about its relationship to aging showed a 20–30% life span extension in mice overexpressing this gene (Kurosu et al. 2005; Kuro-o 2008). The *Klotho* protein exists in two forms: 135-kD membrane *Klotho* and 130-kD secreted *Klotho*. It originally exists as a 135-kD membrane protein where 10 amino acids are located inside the cell, and a 130-kD protein is secreted outside the cell into blood, urine, and CSF through ectodomain shedding (Kuro-o 2011). It is a secreted *Wnt* antagonist that suppresses the *Wnt* signal delivery system, which plays a significant role in embryonic development and tissue restoration. More studies have shown that *Wnt* signaling is abnormally activated in carcinogenesis in various cancers, including prostate cancer (Klaus & Birchmeier 2008). In addition, *KLOTHO* suppresses the *Wnt* signaling system and functions as a tumor suppressor (Liu et al. 2007). A recent study showed that expression of *KLOTHO* is suppressed in cervical cancer (Lee et al. 2010), stomach cancer (Wang et al. 2011), colorectal cancer (Pan et al. 2011), and breast cancer (Rubinek et al. 2012) due to epigenetic regulation.

Epigenetic regulation is defined as having an effect on genetic expression without affecting DNA sequences, and it is involved in gene expression by histone modification, chromatin structure, non-coding RNA and DNA methylation (Skinner et al. 2010; Majumdar et al. 2011; Guerrero-Bosagna & Skinner 2012). DNA methylation is a representative mechanism of epigenetic regulation which refers to the bonding action of the carbon 5 of cytosine in a CpG dinucleotide with guanine. CpG is mostly methylated in the DNA of most vertebrates and is methylated 85% of the time in humans. Unmethylated CpG is usually concentrated in short DNA sequences called CpG islands. CpG islands are located in promoters, suppress gene expression with methylation, and regulate genomic imprinting and X chromosome inactivation. This also promotes the inactivation of anti-oncogenes, which leads to cancer (Gardiner-Garden & Frommer 1987; Ng & Adrian 1999; Bird 2002; Garinis et al. 2002; Takai & Jones 2002; Fujihara et al. 2012).

There are no studies regarding gene expression or epigenetic suppression of *KLOTHO*, although a number of genes – including *Glutathion S-transferase PI (GSTP1)* (Majumdar et al. 2011), *Estrogen Receptor Alpha (ERα), Estrogen Receptor Beta (ERβ) (Sasaki et al.*^200^*2), Adenomatous polyposis coli (APC), RAS association domain family 1A (RASSF1A) (Liu et al.**2011**), Retinoic acid receptor beta 2 (RARB2)* (Gao et al. 2013), and *Prostaglandin-endoperoxide synthase 2 (PTGS2)* (Bastian et al. 2007) – are hypermethylated in prostate cancer. Therefore, this study investigated whether the *KLOTHO* gene is expressed in 22Rv1, DU145, and PC-3, which are human prostate cancer cell lines, and tried to clarify the epigenetic mechanism that regulates gene expression.

## 2. Materials and methods

### 2.1. Cell culture

22Rv1 (hormone-dependent prostate cancer cell line), DU145 and PC-3 (hormone-refractory prostate cancer cell line) were purchased from American Type Culture Collection and used in the study. 22Rv1 was cultured in RPMI1640 (Welgene) medium, DU145 was cultured in MEM (Welgene) medium and PC-3 was cultured in Ham’s F-12 K (Welgene) medium, and 10% FBS and Antibiotic & Antimycotic solution (Welgene) were added in those media. The incubator was maintained at 5% of CO_2_, proper humidity and 37°C.

The expression of KLOTHO mRNA was monitored by treating DU145 and PC-3 cell lines with 0.5, 1, 2, 5, and 10 µM of 2′-deoxy-5-azacytidine (DAC) (Invivogen), a DNA methyltransferase (DNMT) inhibitor, for 72 h or with 1 µM for 12, 24, 28,72, and 96 h. In a separate experiment, we added trichostatin A (TSA) (Sigma), a histone deacetylase, to the DU145 and PC-3 cell lines at a concentration of 25, 50, 100, 250, or 500 nM for 24 h and with a concentration of 100 nM for 6, 12, 24, and 48 h. To monitor the changes in *KLOTHO* expression when each cell line was treated with DAC and TSA, 1 µM DAC was applied for 72 h and 100 nM TSA was applied for 24 h to the DU145 cell line and 1 µM DAC was applied for 96 h and 100 nM TSA was applied for 24 h to PC-3 cell line. Gene expression in each cell line was investigated. The DU145 and PC-3 cell lines without DAC and TSA treatment were used as the control group.

### 2.2. Reverse transcription polymerase chain reaction (RT-PCR)

After extracting the total RNA of all prostate cell lines using an RNeasy Mini Kit (Qiagen), the content and purity of RNA were assessed by measuring the absorbance at 260 and 280 nm with a Nanodrop spectrophotometer (ASP-2680, ACTGene).

The extracted total RNA was used for reverse transcription using ImProm-II™ Reverse Transcription System (Promega). One microgram of the extracted RNA of each cell line, 1 µl of oligo (dT) 15 primer, and 5 µl of nuclease-free water were denatured at 70°C for 5 min. Then, nuclease-free water, ImProm-IITM 5X Reaction Buffer, MgCl2, dNTP Mix, Recombinant RNasin® Ribonuclease Inhibitor (RNasin), and ImProm-IITM Reverse Transcriptase (RTase) were added to 5 µl of this mixture, to a total volume of 20 µl. cDNA synthesis was performed at 25°C for 5 min, at 42°C for 60 min and at 70°C for 15 min.

e mixed 10 pmole/µl of primer and distilled water in AccuPower® PCR PreMix (Bioneer) and performed AccuPower® PCR PreMix (Bioneer). After denaturation at 94°C for 3 min, reactions were cycled 37 times for 30 s at 94°C, 30 s at 68°C and 30 s at 72°C. The sequence and size of the *KLOTHO* (KL) primer is shown in Table 1. Electrophoresis was performed with the amplified PCR product on a 1% agarose gel, gene expression was investigated using the Gel Documentation System (Alpha Innotech), and ACTB (*β*-actin) was used as a housekeeping gene.

**Table 1.** Sequence and characterization of primers used in this study.

Gene		Sequences	Size	TM	Cycles
*ACTB*	Forward	CCATCGAGCACGGCATCGTCACCA	376 bp	68°C	22 cycles
	Reverse	CTCGGTGAGGATCTTCATGAGGTAGT			
*KLOTHO* (*KL*)	Forward	ACTCCCCCAGTCAGGTGGCGGTA	350 bp	68°C	37 cycles
	Reverse	TGGGCCCGGGAAACCATTGCTGTC			
*KL-*U1 (U)	Forward	AGAGGATGTGTGGTAGGTAAAGAG	213 bp	62°C	34 cycles
	Reverse	ACAAACCAAAACTACCTCCACCCT			
*KL-*M1 (M)	Forward	AGAGGACGCGCGGTAGGTAA	213 bp	59°C	
	Reverse	ACGAACCGAAACTACCTCCGC			
*KL-*U2 (U)	Forward	GGTTTGGGTGGTTGTTGTTTGTG	154 bp	62°C	37 cycles
	Reverse	TCTAATAAACAACACTACCCACAACC			
*KL-*M2 (M)	Forward	GGCGGTCGTCGTTTGCGT	144 bp	62°C	
	Reverse	ATAAACGACGCTACCCACGAC			
*KL-*U3 (U)	Forward	GGGGGAATTTTTTTTAGTGTATGGT	188 bp	61°C	33 cycles
	Reverse	TACCAAACCCTAACAAAACACA			
*KL-*M3 (M)	Forward	GGGAATTTTTTTTAGCGTACGGC	184 bp	63°C	
	Reverse	CTACCGAACCCTAACGAAACG			
*KL-*U4 (U)	Forward	GTTAGTGGAGTTTGTTGGGGAGT	186 bp	65°C	36 cycles
	Reverse	AACACCAACAACAACAACAACAACA			
*KL-*M4 (M)	Forward	GTTAGCGGAGTTCGTCGGG	184 bp	63°C	
	Reverse	CCAACAACAACGACAACGACG			
*KL-*BSP	Forward	GGTAATAGTAAAAGGGAGAGTAAAATTT	465 bp	54°C	40 cycles
	Reverse	AAAAAACACCTATTTCTCCCAAC			
*KL*-Pyro	Forward	TGGGTTTTYGAGTGGGAGAAAAGTGA	234 bp	58°C	45 cycles
	Reverse	CACCTATTTCTCCCAACTCC			
	Sequencing	AGTGAGAGTAGGTGT			

### 2.3. Quantitative real-time PCR

After reverse transcription reaction with QuantiTect® Reverse Transcription (Qiagen), we verified the result of RT-PCR and quantified the expression of *KLOTHO* mRNA by performing real-time PCR. We mixed total RNA 1 µg, DNA Wipeout Buffer 2 µl, and nuclease-free water to a total of 14 µl and removed genomic DNA by treating the mixture at 42°C for 2 min. Then, we mixed in RT primer Mix 1 µl, Quantiscript RT Buffer 4 µl, and Quantiscript Reverse Transcriptase QRTase) 1 µl and performed a reverse transcription reaction at 42°C for 15 min. 20 uL cDNA was synthesized by suppressing QRTase at 95°C for 3 min and a quantitative real-time PCR reaction was performed using Rotor Gene Q (Qiagen). The expressed amount of *KLOTHO* mRNA from each cell line was quantified using ACTB as a housekeeping gene.

### 2.4. Bisulfite conversion

**Figure 1.**
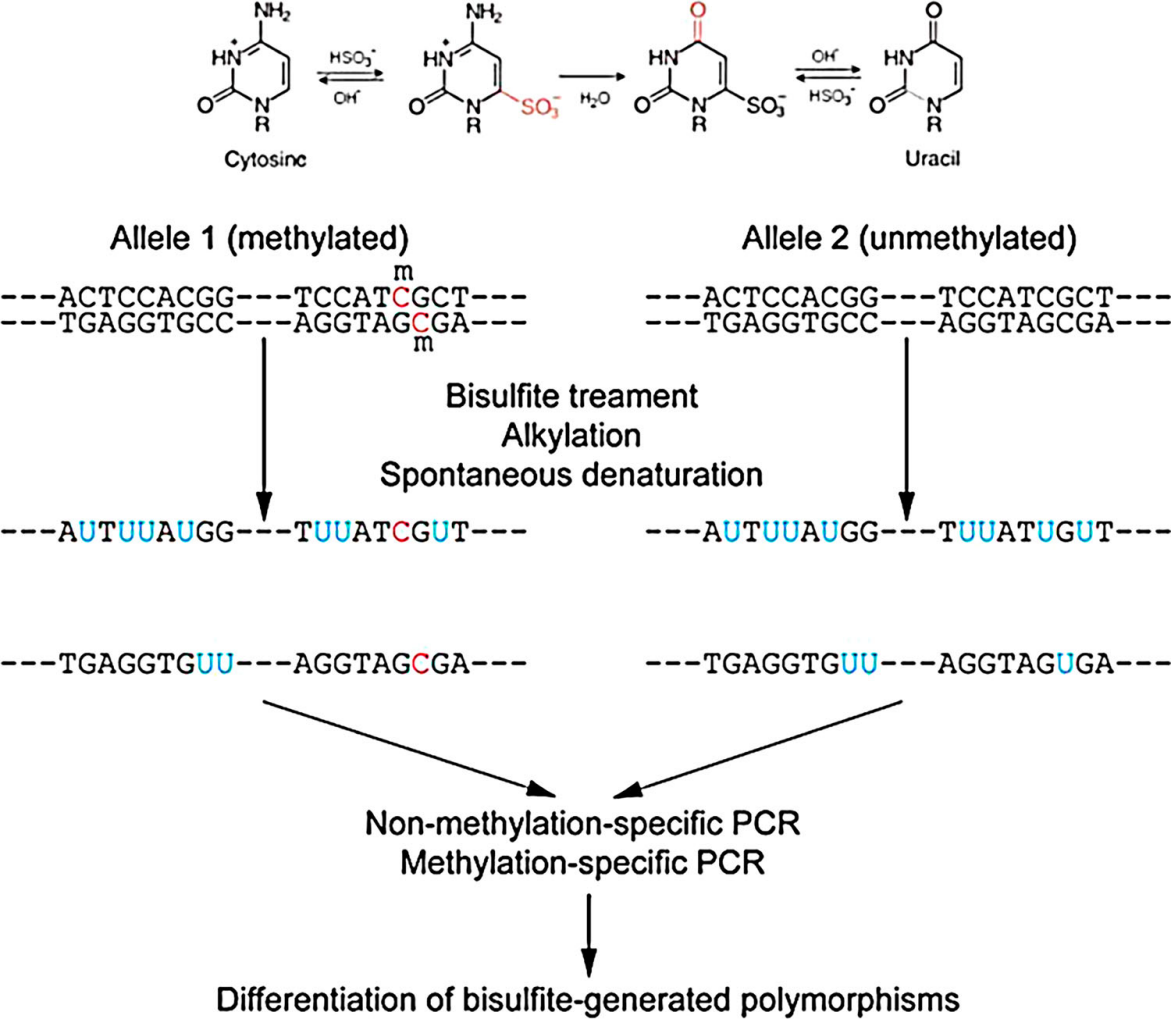
Bisulfite conversion of genomic DNA.

To proceed with our DNA methylation experiments, bisulfite conversion was performed (Figure 1). The genomic DNA of each cell line was extracted using a QIAamp^®^ DNA Mini Kit (Qiagen) and unmethylated cytosines in 2 µg of genomic DNA were switched to uracil using EZ Methylation Kit (Zymo Research).

### 2.5. Methylation-specific PCR (MSP)

We made a 20-µl mixture with 1 µl of the Bisulfite DNA, 2 pmole/µl primers, 10X buffer, MgCl2, HS Prime Taq DNA Polymerase (GeNet Bio) and nuclease-free water, and performed MSP with a thermal cycler (Takara). The mixture was denatured at 94°C for 10 min and cycled as appropriate for each primer, including 30 s at 94°C, 30 s at each primer temperature, 20 s at 72°C, and then 72°C for 10 min. The amplified PCR products were verified by performing electrophoresis with a 2% agarose gel.

### 2.6. Bisulfite genomic sequencing

The genetic sequence of the DNA promoter containing 35 CpG was investigated by amplifying the KL-BSP primer (Table 1). First, we mixed 2 pmole/µl primer, 10X buffer, MgCl2, HS Prime Taq DNA Polymerase (GeNet bio) and nuclease-free water to a total volume of 50 µl and amplified DNA using a thermal cycler (Takara). The mixture was denatured at 94°C for 10 min and cycled 40 times for 30 s at 94°C, 30 s at 54°C, and 40 s at 72°C, followed by 10 min at 72°C. We performed electrophoresis with a 1% agarose gel with the amplified DNA to evaluate the suitability of the DNA. The 465 bp DNA fragments obtained with QIAEX II®Gel Extraction Kit were refined using the DNA Clean & Concentrator™-5 kit (Zymo Research). Each refined DNA was inserted into a T-vector through the pGEM-T Easy Vector system (Invitrogen) and a clone was produced by incubation at 37°C overnight. Each clone was evaluated by colony PCR. Successful clones were investigated for CpG methylation by bisulfite genomic sequencing.

### 2.7. Bisulfite pyrosequencing

We quantified the methylation of 4 CpG sites from 16 to 19 by bisulfite pyrosequencing and mixed 1 µl of the bisulfite DNA with 10X buffer, MgCl2, HS Prime Taq DNA Polymerase (GeNet Bio), and distilled water for a total of 25 µl. This was amplified using a thermal cycler (Takara) with denaturation at 94°C for 10 min followed by 45 cycles of 94°C for 30 s, 58°C for 30 s, and 72°C for 20 s, followed by 72°C for 10 min. The bisulfite pyrosequencing experiment was performed with the amplified DNA product.

## 3. Results

### 3.1. Confirmation of the *KLOTHO* mRNA expression in prostate cell lines

**Figure 2.**
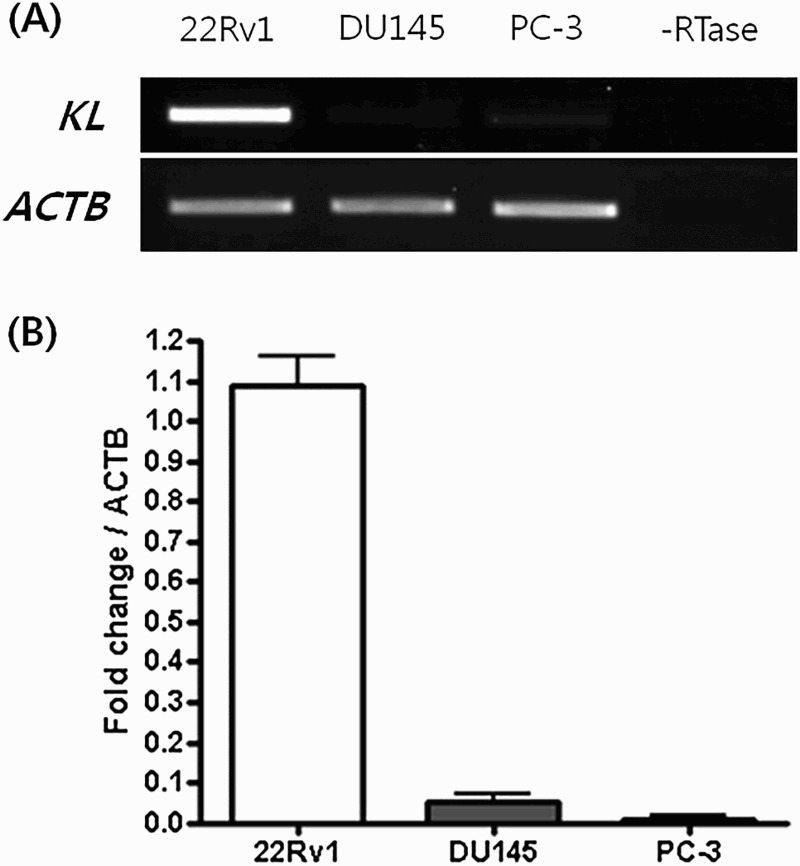
Expression of the KLOTHO mRNA in the prostate cancer cell lines.

RT-PCR shows that *KLOTHO* mRNA expression was activated in the 22Rv1 cell line and suppressed in the DU145 and PC-3 lines. This is concordant with quantitative real-time PCR data on *KLOTHO* mRNA expression in each cell line. The threshold cycle of *KLOTHO* mRNA was 15.13 ± 0.11 in the 22Rv1 cell line, 19.74 ± 1.07 in the DU145 cell line and 19.74 ± 1.07 in the PC-3 cell line. The amount of expression was measured in each cell line based on these figures. Considering the expression of *KLOTHO* mRNA in 22Rv1 as 100, expression in DU145 and PC-3 cell line were only 4 and 1, respectively. That is, *KLOTHO* expression is 1/25 in DU145 and 1/100 in PC-3 compared to 22Rv1 (Figure 2).

### 3.2. Restoration of *KLOTHO* mRNA expression with an epigenetic methyltransferase inhibitor

**Figure 3.**
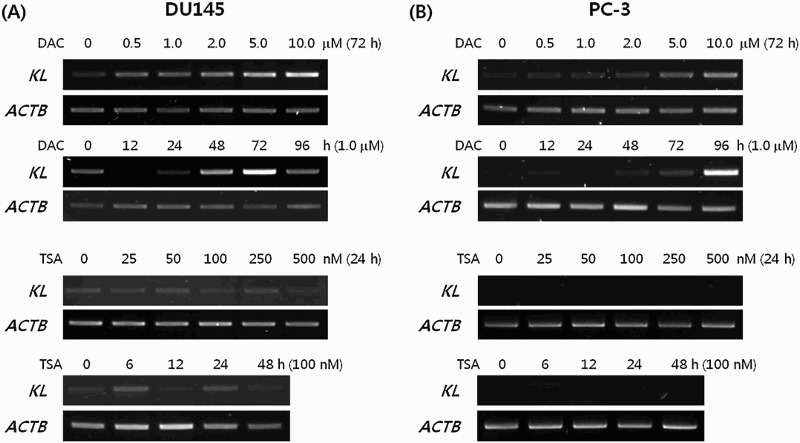
Restoration of the KLOTHO mRNA by treatment with either DAC or TSA in prostate cancer cell lines DU145 and PC-3, respectively.

**Figure 4.**
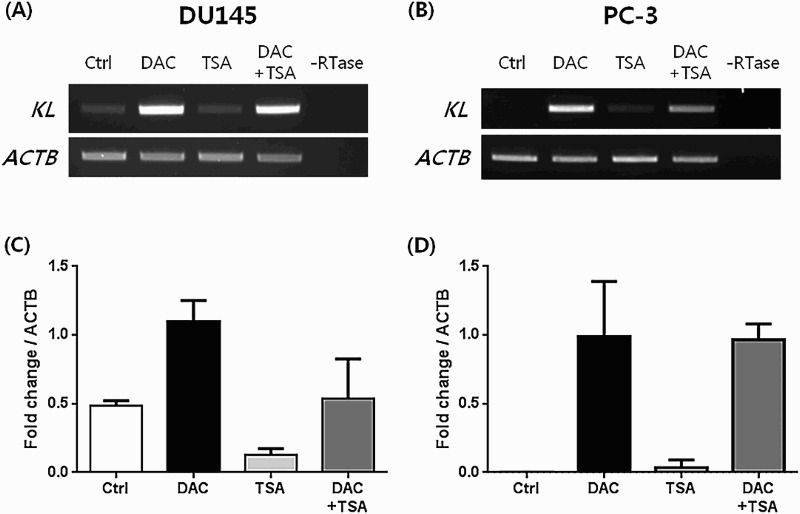
Restoration of the KLOTHO mRNA by treatment with DAC and TSA in prostate cancer cell lines DU145 and PC-3, respectively.

We treated DU145 and PC-3, which do not express *KLOTHO*, with DAC to suppress DNA methylation. RT-PCR showed that expression was restored in a dose- and time-dependent manner after DAC treatment. However, TSA treatment of DU145 and PC-3 did not produce changes (Figure 3(a,b)). *KLOTHO* mRNA expression in DU145 cells decreased more during combined TSA and DAC treatment compared to DAC alone (Figure 4(a)). PC-3 showed no difference between DAC + TSA vs DAC alone, but the level of decrease was lower than seen in DU145 (Figure 4(b)). Quantitative real-time PCR that was performed to verify the RT-PCR result showed a control threshold cycle of 17.89 ± 0.09 in the DU145 cell line, compared to 16.72 ± 0.13 in DAC, 19.89 ± 0.67 in TSA, and 17.91 ± 0.95 with combined DAC and TSA treatment. DAC treatment increased *KLOTHO* mRNA expression two-fold compared to the control, and TSA decreased *KLOTHO* mRNA expression by less than one-third of the control. Combined treatment showed similar expression results (Figure 4(c)). The threshold cycle of *KLOTHO* mRNA in the PC-3 cell line was 27.3 ± 0.08 in the control, 18.42 ± 0.68 in DAC, 25.23 ± 3.57 in TSA, and 18.38 ± 0.18 in DAC + TSA. Therefore, *KLOTHO* mRNA expression was increased 500X by DAC and 10X by TSA compared to the PC-3 cell line control. Combined treatment with DAC and TSA showed no synergistic action and similar expression to DAC treatment alone in the PC-3 cell line (Figure 4(d)).

### 3.3. Methylation analysis of prostate cell lines through methylation-specific PCR (MSP)

**Figure 5.**
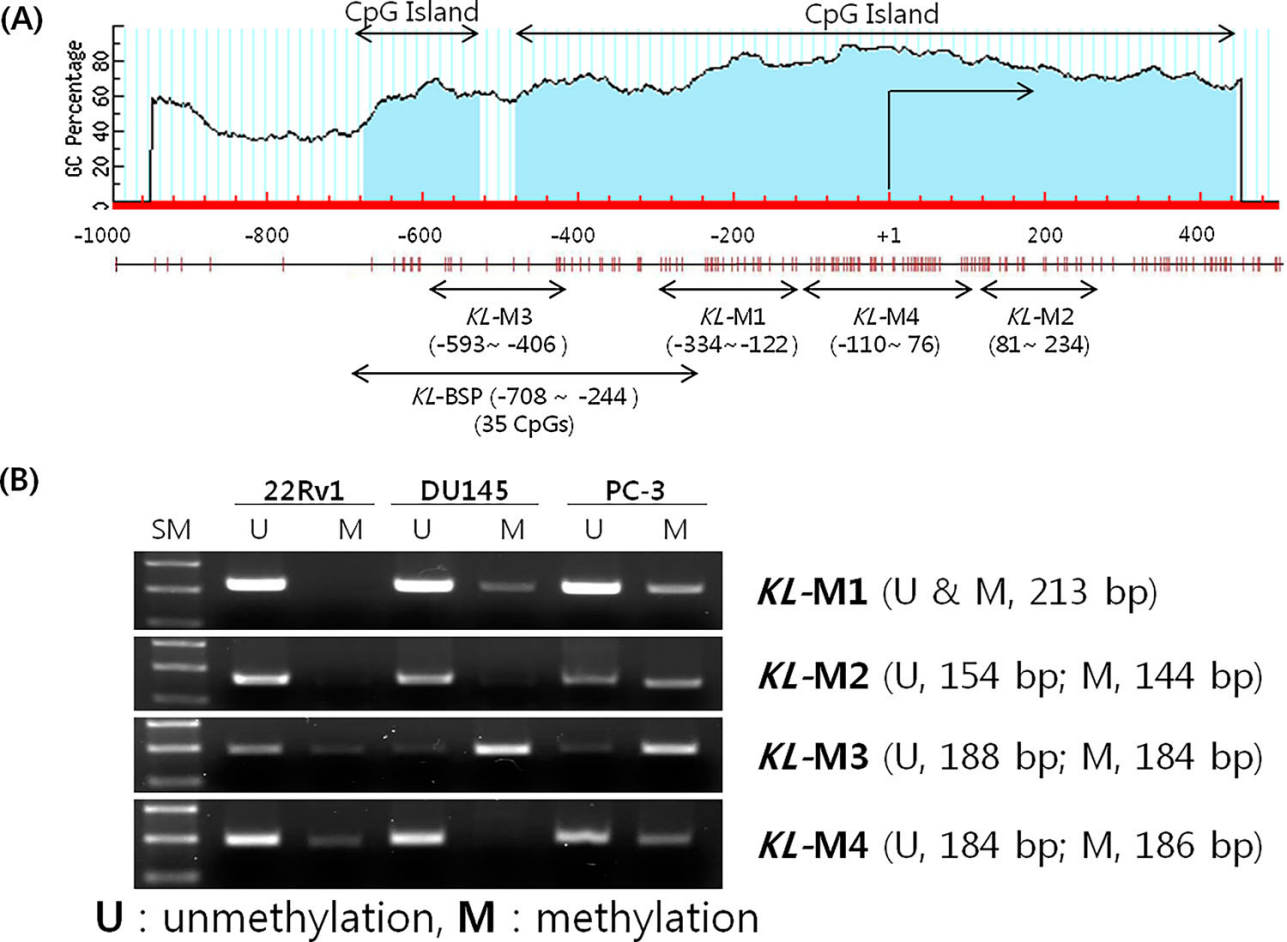
Methylation status of the KLOTHO CpG island region in prostate cancer cell lines.

We investigated the location of CpG islands and CpGs from 500 to 1000 bp in the *KLOTHO* gene using the Methprimer program (http://www.urogene.org/methprimer/index1.html) (Figure 5(a)). Since *KLOTHO* gene suppression with DNA methylation in the DU145 and PC-3 lines was verified through RT-PCR and quantitative real-time PCR, the location of CpG methylation in both cell lines was investigated.

PCR was performed with the four MSP primers produced: the KL-M1 primer amplifying the −334∼ −122 bp area on a CpG island in the *KLOTHO* DNA, the KL-M2 primer amplifying the 81−234 bp area, the KL-M3 primer amplifying the −593 ∼−406 bp area, and the KL-M4 primer amplifying the −110 ∼ 76 bp area. The amplified product from the KL-M3 primer in the 22Rv1 cell line, which expresses *KLOTHO* mRNA, showed stronger amplification of the unmethylation (U) primer, while the DU145 and PC-3 cell lines, which do not express *KLOTHO* mRNA, showed only the amplified product of the methylation (M) primer (Figure 5(b)).

### 3.4. Methylation analysis of CpG site through Bisulfite genomic sequencing

**Figure 6.**
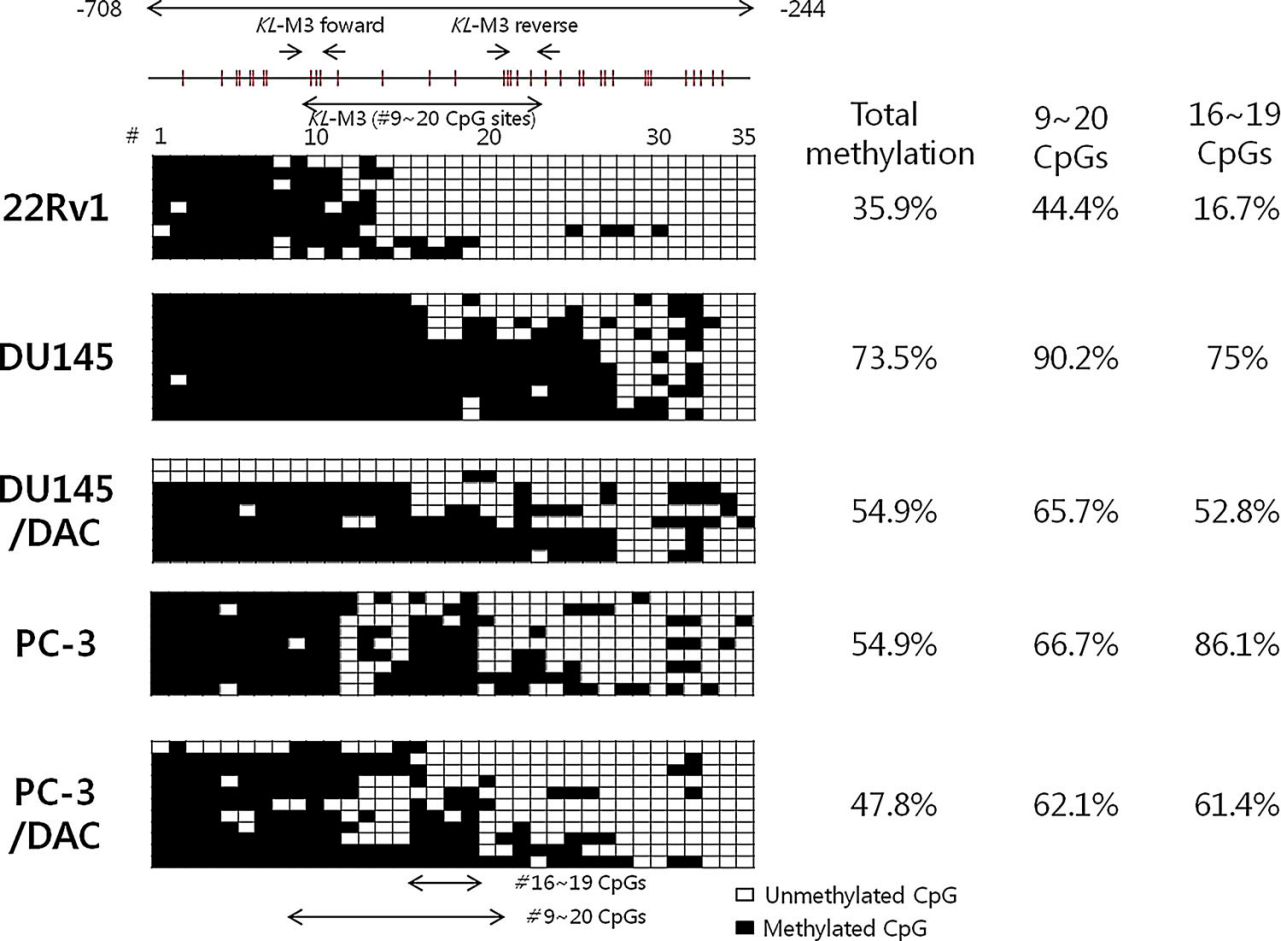
Bisulfite sequencing analysis of the KLOTHO promoter region in the prostate cancer cell lines.

Bisulfite genomic sequencing of DNA from −708 to −244 bp in the *KLOTHO* gene promoter was performed to investigate the base sequence of DNA including 35 CpG. The methylation ratio was calculated by comparing the number of entire CpGs and methylated CpGs. The 22Rv1 cell line showed 35.9% methylated CpGs, while the DU145 and PC-3 cell lines showed 73.5% and 54.6% methylated CpGs, respectively. The methylation ratios of the DU145 and PC-3 cell lines were 54.9% and 47.8%, respectively, and decreased with DAC treatment. The ratio of 9–20 CpGs was 44.4% in the 22Rv1 cell line, 90.2% in the DU145 cell line, and 66.7% in the PC-3 cell line. DAC treatment led to a ratio of 67.5% and 62.1% in the DU145 and PC-3 cell lines, respectively. The area which showed the most obvious change was CpG-16 to -19. Only 16.7% of those were methylated in the 22Rv1 line while 75% were methylated in DU145 and 86.1% were methylated in PC-3. In addition, DAC treatment decreased methylation more than 20% in the DU145 and PC-3 lines to 52.8% and 61.4%, respectively (Figure 6).

### 3.5. Methylation analysis of CpG site through Bisulfite pyrosequencing

**Figure 7.**
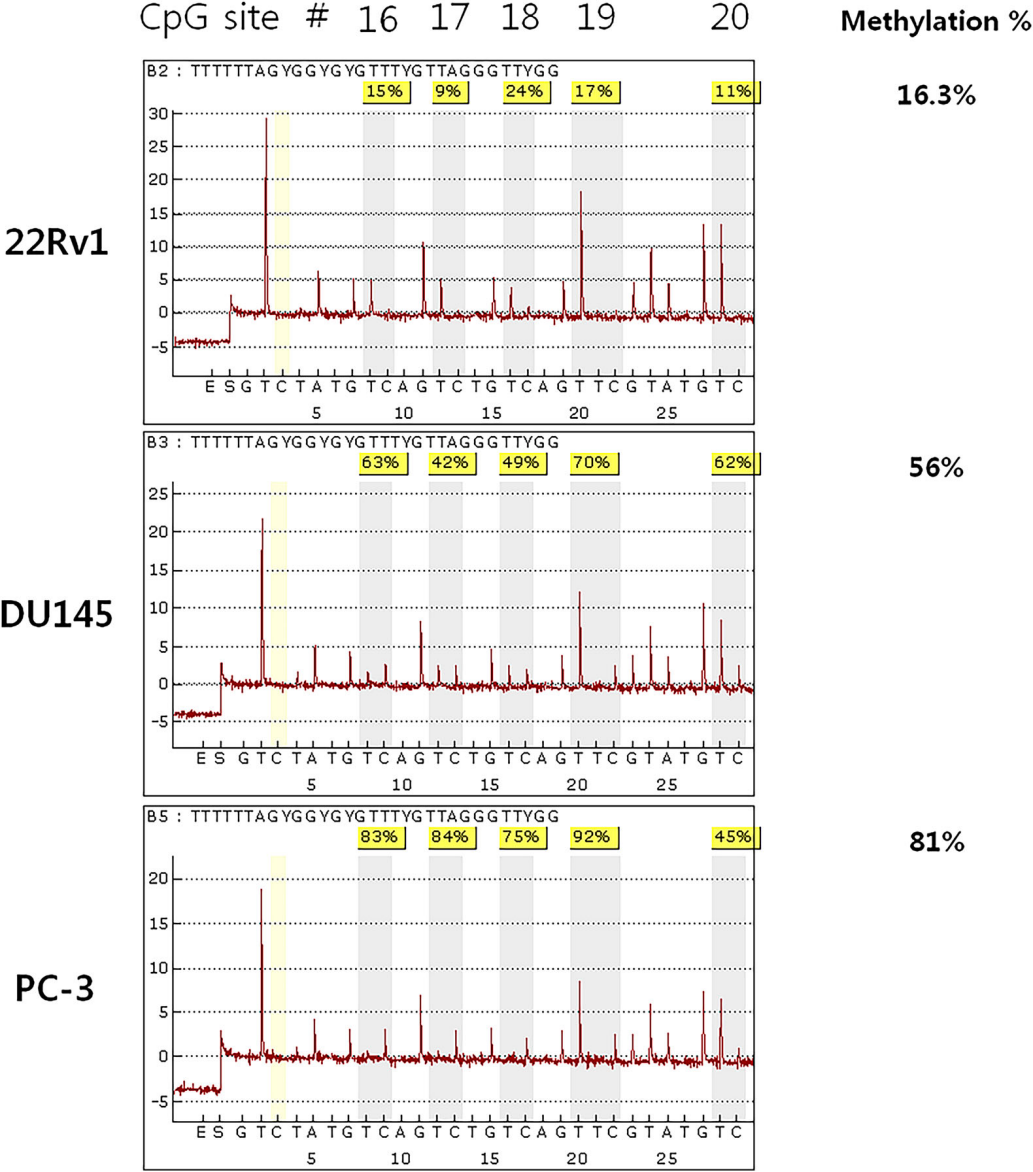
Bisulfite pyrosequencing analysis of the KLOTHO promoter region in the prostate cancer cell lines.

The methylation ratio was verified with bisulfite genomic sequencing and quantified with bisulfite pyrosequencing. The result showed that the ratios of CpG-16 to CpG-19 were 15%, 9%, 24%, and 17% respectively, in the 22Rv1 cell line, with an average of 16.3%. The U145 and PC-3 lines, which showed suppression of *KLOTHO* mRNA, showed methylation ratios of 63%, 42%, 49%, and 70% with an average of 56%, while the PC-3 line showed methylation ratios of 83%, 84%, 75%, and 92% with an average of 81% (Figure 7).

## 4. Discussion

As the incidence of prostate cancer in men has increased sharply over the past 10 years in Korea, it is a growing public health concern (Jung et al. 2015). Although prostate cancer mainly occurs in the elderly, the exact connection between aging and prostate cancer has not been investigated clearly.

*KLOTHO* is an anti-aging gene and previous studies showed that *KLOTHO*-deficient-mice have a shorter life span, with a similar a phenotype to aging in humans (Kuro-o et al. 1997). *KLOTHO* also plays a role as a secreted Wnt antagonist, suppressing cancer (Liu et al. 2007). It is expressed mainly in the kidney and brain (Kuro-o et al. 1997), and expression was also observed in the cervix (Lee et al. 2010), stomach (Wang et al. 2011), large intestine (Pan et al. 2011), and breast (Rubinek et al. 2012). We demonstrated that the *KLOTHO* mRNA is expressed in prostate cell lines for the first time by identifying expression in the 22Rv1 prostate cell line. However, the DU145 and PC-3 prostate cell lines did not show expression (Figure 2). The *KLOTHO* mRNA expression of these two cell lines was recovered when each cell line was treated with DAC, while it remained unchanged with TSA treatment (Figure 3). There was a similar or lower level of restoration in expression with combined DAC and TSA treatment compared to DAC alone (Figure 4). This indicates that expression of *KLOTHO* mRNA in DU145 and PC-3 is suppressed only by DNA methylation, and that changes in histones do not strongly affect *KLOTHO* mRNA expression.

An MSP reaction which was performed to identify CpG methylation affecting *KLOTHO* mRNA expression showed that KL-M3, including −593 to −406 bp, was methylated in the DU145 and PC-3 cell lines. These lines show suppressed expression of *KLOTHO* mRNA due to DNA methylation. This segment was unmethylated in the 22Rv1 cell line, which shows expression of *KLOTHO* (Figure 5). Therefore, methylation of CpGs from −593 to −406 bp in the *KLOTHO* promoter regulates the expression of *KLOTHO* mRNA.

Bisulfite genomic sequencing was performed on the *KLOTHO* gene promoter with a KL-BSP primer, which included KL-M3 (from −59 to −406 bp). The results showed that the *KLOTHO* promoter was hypomethylated in the 22Rv1 cell line and hypermethylated in the DU145 and PC-3 cell lines. Four CpGs from CpG-16 to CpG-19 showed 16.7% methylation in the 22v1 cell line, 75% in the DU145 cell line, and 86.1% in the PC-3 cell line. In addition, the methylation ratio in the DU145 and PC-3 cell lines decreased by 20% with DAC treatment (Figure 6). Therefore, methylation of CpG-16 to CpG-19 in the *KLOTHO* gene promoter regulates the expression of *KLOTHO* mRNA in prostate cell lines.

Bisulfite pyrosequencing was analyzed to digitize the DNA methylation density of four CpGs which were identified by bisulfite genomic sequencing. CpG-16 to CpG-19 were 16.3% methylated in 22Rv1 while the DU145 and PC-3 cell lines were 56% and 81% methylated, respectively (Figure 7). This result demonstrated an inverse relationship between CpG methylation density and expression of *KLOTHO* mRNA. This is consistent with our data showing that *KLOTHO* mRNA is expressed during promoter hypomethylation and not in hypermethylation (Lee et al. 2010; Pan et al. 2011; Wang et al. 2011; Rubinek et al. 2012).

This study is the first to demonstrate suppression of *KLOTHO* mRNA expression due to promoter DNA methylation. However, it is limited by the fact that the experiments in this study were not performed with tissue from prostate cancer patients. Thus, further research is needed to investigate whether *KLOTHO* mRNA is expressed in patient tissue and whether it is suppressed due to DNA methylation.

## 5. Conclusion

This is the first study demonstrating that the anti-aging gene *KLOTHO* is expressed in prostate cancer cell lines. We have shown the relationship between *KLOTHO* mRNA expression and DNA methylation in human prostate cell lines. We confirmed that *KLOTHO* mRNA is expressed in 22Rv1, a human prostate cell line, while it is suppressed in DU145 and PC-3 due to DNA methylation, showing an inverse correlation between the expression of *KLOTHO* mRNA and DNA methylation.
